# Structural characteristics of local cortical networks wired by distance dependent connectivity rules

**DOI:** 10.1186/s12868-026-01050-1

**Published:** 2026-09-19

**Authors:** Bernhard Hellwig

**Affiliations:** Rempartstr. 11, 79098 Freiburg, Germany

**Keywords:** Cerebral cortex, Pyramidal neuron, Local connectivity, Connection probability, Distance dependent, Neuronal assembly, Network science

## Abstract

**Background:**

The function of the cerebral cortex is shaped by its anatomical connectivity, yet experimental findings on connection probabilities in local cortical networks remain inconsistent. This study explores structural characteristics of local cortical networks based on distance dependent, Gaussian connectivity profiles. Monolayers of 101 × 101 pyramidal neurons were examined. Their connectivity was based on experimental anatomical or electrophysiological data. In the anatomical setting the connection probability between neighboring neurons was 0.8. In the electrophysiological scenario connection probabilities for adjacent neurons ranged between 0.08 and 0.23. All distance dependent networks were compared to the configuration model which generates degree-preserving but otherwise randomly rewired networks. The networks thus constructed were analyzed applying tools of network science, i.e. average degrees, degree distributions, local clustering coefficients and graph distances. Moreover, the numbers, sizes and spatial dimensions of cliques were investigated as well as the cost of connectivity.

**Results:**

Distance-dependent networks differed fundamentally from configuration-model networks across all structural measures. They showed substantially higher local clustering, formed more numerous and more spatially compact groups of strongly connected neurons, and required lower wiring cost. Importantly, the structure of distance-dependent networks was highly sensitive to near-neighbor connectivity: when neurons had a high probability of connecting locally, the network reliably developed tightly wired, spatially localized neuronal clusters.

**Conclusions:**

Distance-dependent connectivity gives rise to structural network features that may facilitate the emergence of functional neuronal assemblies. Based on the findings of this study, a general probabilistic rule for local cortical connectivity is proposed that can be used to design artificial neural networks with biologically inspired wiring principles.

## Background

To build a neural network in its most elementary form, two principal network characteristics must be known: the number of neurons and the rules by which these neurons are connected. Much of the wiring in the brain seems to be innate, the crystallization of a long evolutionary process [1, 2]. Zador [2] argued that the rules for wiring up the brain must be simple, since there is a “genomic bottleneck”: the connectivity in the brain is far too complex to be specified in detail by the genes. Nevertheless, the innate wiring seems to be powerful. Animals and humans may show complex behavior immediately after birth, before learning processes have occurred to a relevant degree.

In the cerebral cortex, it is challenging to determine the rules of connectivity. The reason is that different spatial scales must be observed simultaneously. While the diameter of synapses is on the order of 1 μm, neuronal ramifications may extend over millimeters or centimeters. There are no simple instruments to disentangle structures whose key components differ in size by factors of 10^3^ to 10^4^. Unsurprisingly, experimental findings concerning local connectivity in the cortex were heterogeneous and quite controversial, as reviewed below.

Based on statistical and geometrical considerations of cortical anatomy, Braitenberg and Schüz [1] argued that the probability of a connection between adjacent pyramidal neurons is high between 0.63 and 0.87 while connectivity for neurons further apart is sparse and weak, mostly mediated by a single synapse. Hellwig [3] and van Pelt and van Ooyen [4] gave an estimate for cortical connectivity by calculating spatial appositions between axonal and dendritic arborizations of pyramidal neurons. Spatial touches between neurons were assumed to be a good estimate for actual synaptic contacts. Connectivity between cortical neurons was distance dependent, the connection probability being around 0.8 for adjacent neurons and falling off with increasing distance between neurons in a Gaussian manner. Lübke et al. [5] actually observed cortical connectivity on different spatial scales by studying light and electron microscopy of the same neurons. They showed that the probability of forming autapses, i.e. synapses of a neuron with itself, is 0.8 for pyramidal neurons in layer 5 of rat somatosensory cortex. Kasthuri et al. [6] and Motta et al. [7] provided dense reconstructions of cortical tissue using serial electron microscopy, so far, however, restricted to volumes much smaller than the extent of axonal and dendritic trees.

Due to the challenges of describing cortical connectivity by anatomical methods, connection probabilities in local cortical networks were predominantly studied using electrophysiology. Functional connectivity between two or more neurons can be investigated by stimulating a neuron and recording from adjacent neurons. Connection probabilities thus elucidated are much lower than assumed from anatomical studies. In mouse and rat cortex, connection probabilities between pyramidal or spiny stellate neurons within a given layer ranged between 0.07 and 0.26 in layers 2/3 [8–14] between 0.04 and 0.36 in layer 4 [10, 15, 16, 17, 18], between 0.05 and 0.19 in layer 5 [10, 19–22] and between 0.03 and 0.09 in layer 6 [10, 23, 24]. In some electrophysiological studies [25–27], local cortical connectivity was also described as distance-dependent, the connection probability tailing off with increasing distance between neurons. In recent large-scale reconstructions of the cortex the connection probabilities assumed were shaped by the results of electrophysiological studies [28–30].

The experimental findings on local cortical connectivity reported above are so heterogeneous that they cannot all be equally accurate. It must certainly make a difference for network structure and dynamics whether a neuron is connected to 80% of its neighbors or only to 3%. In the present study, experimental findings on local cortical connectivity were taken at face value and used to reverse-engineer artificial neural networks with cortex-like architectures. Structural characteristics of these networks were analyzed and compared. The aim of this study was to identify which of the heterogeneous, experimentally derived connectivity rules yield the most beneficial network properties.

The artificial networks generated in this study consisted of monolayers of 101 × 101 pyramidal neurons. Peter’s rule applied, i.e. connections were considered as non-specific [1, 31, 32]. Networks wired according to electrophysiologically obtained connectivity rules were compared to networks inspired by anatomical data. In the electrophysiological scenario, the results on distance dependent connectivity provided by Holmgren et al. [25], Perin et al. [26] and Levy and Reyes [27] were used. The anatomical scenario applied the result by Lübke et al. [5] who showed that the probability of autapses, i.e. of synapses of a neuron with itself, is 0.8. Ten different Gaussian probability distributions were generated in which the connection probability for distance 0 was always 0.8 while the standard deviations, i.e. the widths of the Gaussians, were systematically altered.

Both the networks in the electrophysiological and the anatomical scenarios were compared to the configuration model [33, 34]. The configuration model generates networks which preserve the number of incoming and outgoing connections (the in-degrees and out-degrees) of each neuron in distance-dependent networks. Otherwise, connections are randomly rewired. In other words, the spatial relations between neurons which are key in distance-dependent networks are disregarded in the configuration model. Thus, the importance of Euclidean distances between neurons for the network structure can be evaluated. For each network based on distance dependent rules a matching network generated by the configuration model was investigated.

All networks thus constructed were analyzed with tools of network science [34], calculating average degrees (average numbers of connections per neuron), degree distributions, local clustering coefficients and graph distances. The numbers, sizes and spatial extensions of cliques, i.e. ensembles of neurons with full connectivity, were calculated. Moreover, the cost of connectivity in the different networks was estimated.

## Methods

All calculations were done in Mathematica 13.2 on an Apple MacBook Pro.

### Setup of networks

The networks investigated here consisted of quadratic monolayers of 101 × 101 = 10,201 pyramidal neurons. Pyramidal neurons are the most frequent cell type in the cerebral cortex accounting for about 85% of all cortical neurons [1]. Neurons were arranged in a regular lattice, i.e. distances between neurons were not random, but evenly spaced. The distance between two immediately adjacent neurons side-by-side or top to bottom was standardized as the base unit 1. Euclidean distances between neurons were calculated using Pythagoras’ theorem and expressed in dimensionless units.

Distance dependent connectivity profiles were assumed to be Gaussian as reported by Hellwig [3]. The following formula was used:

  

1 $$\:{\mathrm{p}=\mathrm{a}\:\mathrm{e}}^{\frac{{-\mathrm{x}}^{2}}{{2\mathrm{c}}^{2}}}$$

where * p* is the connection probability, * a* the connection probability at distance 0, * x* the Euclidean distance between neurons and * c* the standard deviation.

**Fig. 1:**
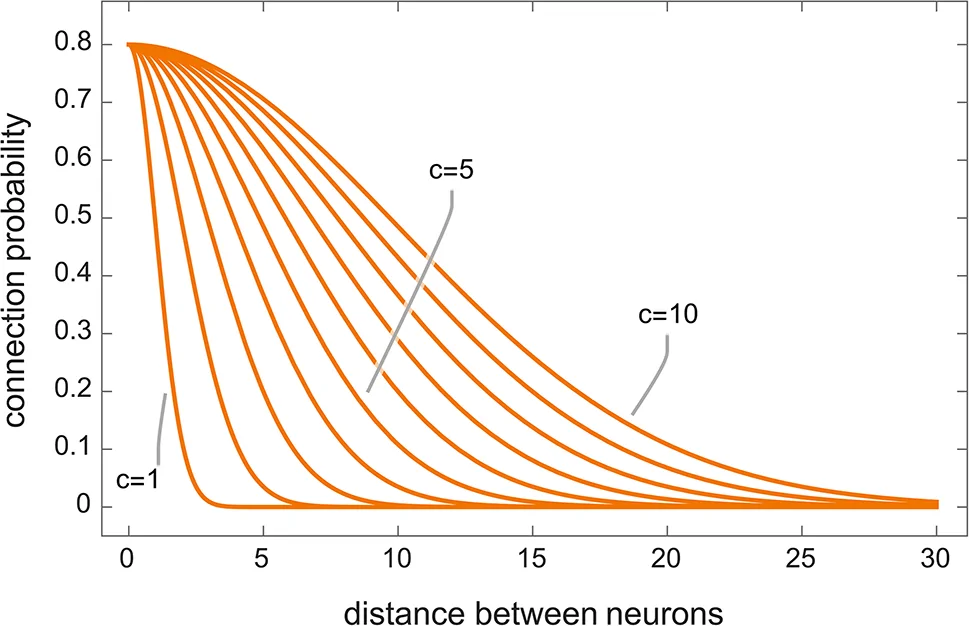
Distance dependent connectivity profiles in the anatomical scenario. The connection probability is a = 0.8 at distance 0 between neurons. For larger distances, connection probabilities fall off in a Gaussian manner, the standard deviations c of the Gaussians varying between 1 and 10

The rules of wiring were motivated by both anatomical and electrophysiological data. In the anatomical scenario, the connection probability for distance 0 models autapses, i.e. synapses of a neuron with itself. In line with findings in neocortical layer 5 pyramidal cells, where approximately 80% of neurons possess autapses [5], the connection probability at zero spatial displacement was set to 0.8 in the anatomical scenario. The standard deviation c of the Gaussians was systematically altered utilizing integers between 1 and 10. The distance dependent connectivity profiles thus obtained are illustrated in Fig. 1.

In the electrophysiological scenario results for distance dependent connectivity by Holmgren et al. [25], Perin et al. [26] and Levy and Reyes [27] were used. In these studies, connectivity was mapped by electrically stimulating one neuron and by simultaneously recording from one or more neighboring neurons. Holmgren et al. [25] studied pyramidal neurons in layers 2/3 of rat visual and somatosensory cortex, the data used here was taken from their Fig. 2D. Perin et al. [26] investigated pyramidal neurons in layer 5 of rat somatosensory cortex, distance dependent connection probabilities were shown in their Fig. 1E. The precise values for the connection probabilities in Perin et al. [26] were specified in Table S2 of the supplemental information of Udvary et al. [30]. Here, values given for the median were used. Levy and Reyes [27] studied neurons in layers 2/3 and 4 of mouse auditory cortex, distance dependent connection probabilities between pyramidal neurons were presented in their Fig. 4A. The authors presented also a Gaussian fit to the data with a connection probability for Euclidean distance 0 of 0.09 and a standard deviation c of the Gaussian of 114 μm.

**Fig. 2:**
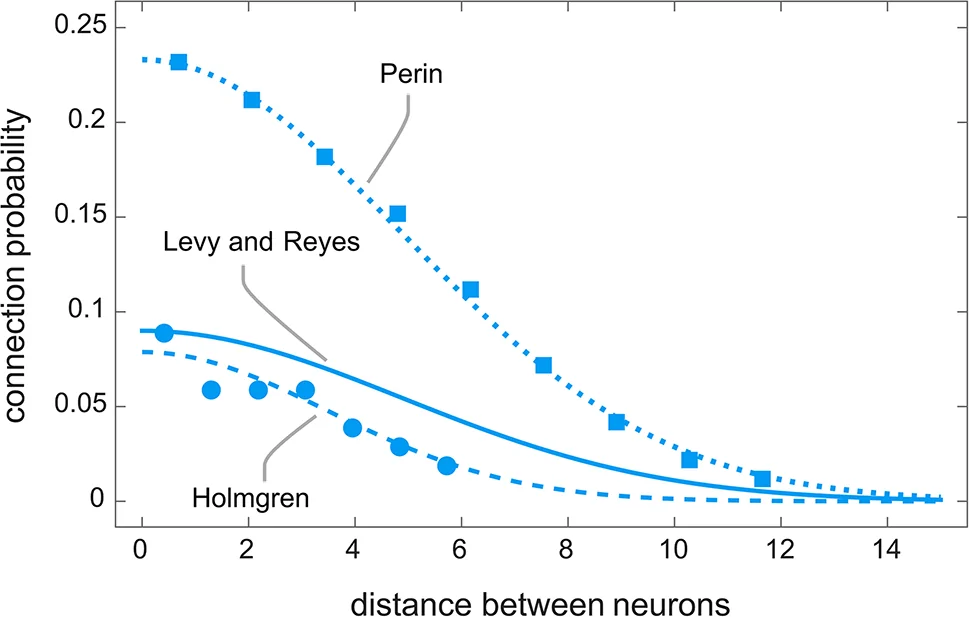
Data for distance dependent connection probabilities from Holmgren et al. [25], Perin et al. [26] and Levy and Reyes [27]. Distances between neurons are displayed in dimensionless units. They are obtained by dividing distances in µm provided by the above studies by the smallest distance between pyramidal neurons given in Table 1. For Holmgren et al. [25] and Perin et al. [26] the standardized data for specific distances and their Gaussian fits are shown (Holmgren: filled dots and dashed line; Perin: filled squares and dotted line). For Levy and Reyes [27], the Gaussian fit provided by the study was used (solid line). Parameters defining the Gaussian connectivity profiles are presented in Table 2. Note that the scales in Fig. 2 are not identical to those in Fig. 1

The data taken from the above studies were standardized such that the smallest distance between pyramidal neurons was the base unit 1. To this end, distances specified in these studies were divided by the smallest distance between pyramidal neurons in µm. This number can be computed by using the volume density of neurons per mm^3^, a number which is generally well known. Results for the specific pieces of cortex investigated by Holmgren et al. [25], Perin et al. [26] and Levy and Reyes [27] are presented in Table 1.

**Table 1:** Density of cortical pyramidal neurons

	Rat somatosensory cortex, layers 2/3	Rat somatosensory cortex, layer 5	Mouse cortex
Number of pyramidal neurons/mm^3^	86,496	60,019	78,455
Number of pyramidal neurons along a line of 1 mm length	44.22	39.15	42.81
Smallest distance between pyramidal neurons (µm)	22.61	25.54	23.36

The number of pyramidal neurons/mm3 was calculated by multiplying overall volume densities of neurons/mm^3^ with 85%, the percentage of pyramidal neurons in the cortex [1]. Overall volume densities of neurons/mm^3^ were based on averages of the following studies: Meyer et al. [35] and Markram et al. [28] for layers 2/3 and layer 5 of rat somatosensory cortex; Schüz and Palm [36] and Keller et al. [37] for mouse cortex. If a study provided data for sublayers, the average weighted according to the thickness of the sublayers was calculated. In Keller et al. [37] the total average of all studies in table 1 was used. The number of pyramidal neurons along a line of 1 mm length is the cube root of the number of pyramidal neurons/mm^3^. The smallest distance between pyramidal neurons can be obtained as the ratio between 1000 μm and the number of pyramidal neurons along a line of 1 mm length

The standardized data for distance dependent connection probabilities in Holmgren et al. [25], Perin et al. [26] and Levy and Reyes [27] are illustrated in Fig. 2. For Holmgren et al. [25] and Perin et al. [26], the Gaussian fits to the data are also shown. For Levy and Reyes [27], the Gaussian fit provided by their study was used. The precise values underlying the electrophysiological scenario are given in Table 2.

**Table 2:** Parameters of the Gaussian connectivity profiles in the electrophysiological scenario

	a (connection probability at distance 0)	c (standard deviation of the Gaussian)
Holmgren et al. [25]	0.079	3.48
Perin et al. [26]	0.233	4.89
Levy and Reyes [27]	0.090	4.88

Values for a (connection probability at distance 0) and c (standard deviation) in the Gaussian connectivity profiles used in the electrophysiological scenario. The connectivity profiles are illustrated in Fig. 2

Using the 13 connectivity profiles in Figs. 1 and 2, computer experiments were performed which yielded 13 connectivity matrices for the quadratic monolayers of 101 × 101 pyramidal neurons. The formation of autapses, i.e. connections of a neuron with itself, were allowed, since autapses do exist in real cortical networks [5]. Due to edge effects neurons at the border of the monolayers form less connections than those in the middle of the monolayers. This was accepted since the cerebral cortex is indeed - at least to some extent - parcellated into areas [e.g. 38], i.e. edge effects are a real feature of cortical anatomy.

For each of the 13 networks based on distance dependent rules a matching network generated by the configuration model [33, 34] was investigated. The configuration model creates networks which preserve the number of incoming and outgoing connections (the in-degrees and out-degrees) of each neuron in distance-dependent networks. Otherwise, connections are random. This was achieved by cutting the connections in distance-dependent networks, shuffling the postsynaptic neurons in a random manner, and rewiring the network. Thus, the spatial relations between neurons which heavily influence the connectivity in distance-dependent networks are disregarded in the configuration model.

All the networks investigated here were directed. More precisely, the out-degree of neurons was studied, i.e. the connections from a given neuron to other neurons.

The datasets generated and analyzed in the current study are available in the zenodo repository [39].

### Analysis of networks

All networks were analyzed using the tools of network science [34]. Average degrees (the average numbers of connections per neuron), degree distributions, local clustering coefficients and graph distances were calculated. Moreover, cliques were studied, i.e. ensembles of neurons in which each neuron is connected by a directed connection to any other neuron of the ensemble. Only maximal cliques were considered, i.e. the cliques were not subsets of larger cliques and could not be extended by adding another neuron. The number of connections dependent on the Euclidean distance between neurons was also investigated. Finally, the costs of the different connectivity matrices were estimated based on the number and the length of connections.

### Network metrics and mathematical definitions

To quantify the structural properties of the directed network models $\:G=\left(V,E\right)$ with $\:N=\left|V\right|$ vertices (neurons) and $\:M=\left|E\right|$ directed edges (where * g* represents the graph in Wolfram Mathematica), formal definitions were applied as follows:

#### *Clustering coefficient*

For a vertex $\:{v}_{i}$, the local directed clustering coefficient $\:{C}_{i}$ measures the proportion of directed edges that exist between its immediate neighbors.

$$\:{C}_{i}=\frac{{e}_{i}}{{k}_{i}^{\mathrm{in}}{k}_{i}^{\mathrm{out}}-{k}_{i}^{\leftrightarrow\:}}$$

where $\:{e}_{i}$ is the number of directed links between neighbors of $\:{v}_{i}$, $\:{k}_{i}^{\mathrm{in}}$ and $\:{k}_{i}^{\mathrm{out}}$ are its in- and out-degrees, and $\:{k}_{i}^{\leftrightarrow\:}$ is the number of reciprocal links. The mean clustering coefficient $\:\stackrel{-}{C}=\frac{1}{N}\sum\:_{i=1}^{N}{C}_{i}$ was computed in Wolfram Mathematica using MeanClusteringCoefficient[g].

#### *Graph distance*

The directed distance $\:d\left(i,j\right)$ is the minimum number of directed edges traversed to travel from vertex $\:{v}_{i}$ to vertex $\:{v}_{j}$. Pairwise distances were computed in Wolfram Mathematica using GraphDistanceMatrix[g].

#### *Maximal cliques*

A maximal clique $\:C\subseteq\:V$ is a fully connected, reciprocal subgraph that cannot be expanded by adding another mutually connected vertex.

$$\eqalign{ & {{\cal C}_{{\rm{max}}}}\left( G \right) = {\rm{\{ }}C \subseteq V\;{\rm{|}}\;\forall \,u,v \in C \cr & \left( {u \ne v \Rightarrow \left( {u \to v} \right) \in E \wedge \left( {v \to u} \right) \in E} \right), \cr & {\rm{and}}\,C\,{\rm{is\,maximal}}\} \cr}$$

Maximal cliques were extracted in Wolfram Mathematica using FindClique[g, Infinity, All].

#### *Cost of connectivity (wiring cost)*

For 2D spatial coordinates $\:{\mathrm{r}}_{i}=\left({x}_{i},{y}_{i}\right)$, total wiring cost * W* was defined as the sum of Euclidean lengths over all directed edges $\:\left(i\to\:j\right)\in\:E$

$$W = \mathop \sum \limits_{\left( {i \to j} \right) \in E} \sqrt {{{\left( {{x_i} - {x_j}} \right)}^2} + {{\left( {{y_i} - {y_j}} \right)}^2}}$$

The results presented here are based on single network realizations for each of the 26 probability distributions. To assess reproducibility, these single-trial results *( x)* were compared against 10 additional, consecutive network realizations. For each set of 10 consecutive trials, the mean ($\:\mu\:$) and standard deviation (* s*) were calculated.

The single trial’s relative deviation (* d*, in %) from the mean was evaluated:

$$\:d=\frac{x-\mu\:}{\mu\:}\times\:100$$

Additionally, the z-score was calculated:

$$\:z=\frac{x-\mu\:}{s}$$

### A note on nomenclature

In the present paper, expressions derived from neuroscience and network science were mixed in a systematic way. As to the setup of the networks, the terminology used in neuroscience was preferred, i.e. “neurons” and “connections” instead of “nodes/vertices” and “edges”. For network analysis, expressions from network science were employed, e.g. “degree” instead of “number of connections per neuron”. However, care was taken to explain the terminology of network science in the neuroscientific context.

## Results

### Average degree and degree distribution

**Fig. 3:**
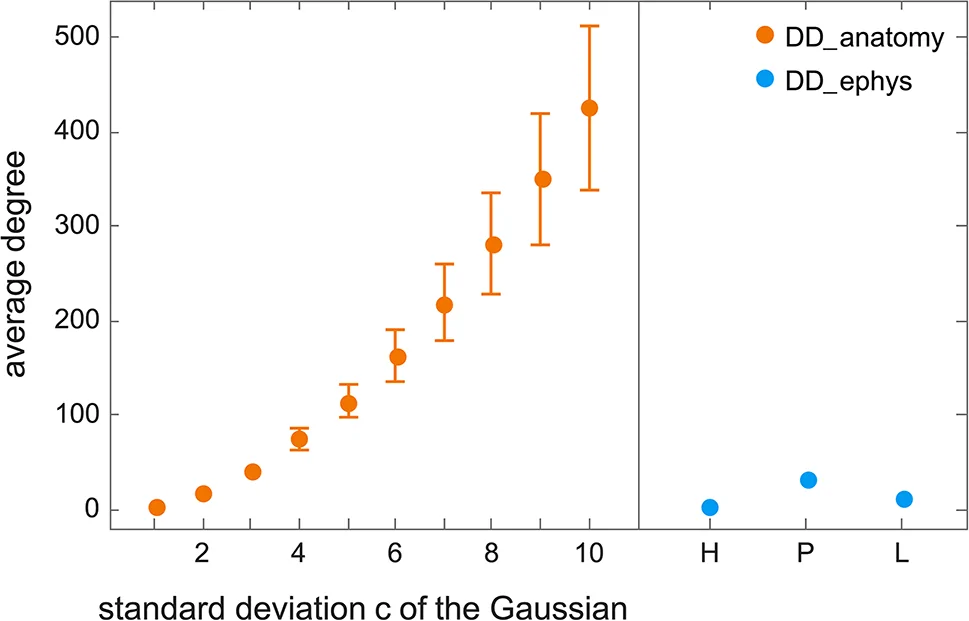
Average degrees for distance dependent networks are displayed in orange in the anatomical scenario (DD_anatomy) and in blue in the electrophyiological scenario (DD_ephys). The label “standard deviation c of the Gaussian” refers to the connectivity profiles in Fig. 1. The letters H, P and L are abbreviations for the studies by Holmgren et al. [25], Perin et al. [26] and Levy and Reyes [27]. Standard deviations are only shown if they are larger than the diameter of the dots

Figure 3 illustrates the average degrees, i.e. the average numbers of connections per neuron, in 13 distance dependent networks. Results for the corresponding configuration models in which the spatial relations between neurons are disregarded are identical and not shown here.

In the anatomical scenario, there was a disproportionate increase of the average degree with increasing standard deviation c of the Gaussian connectivity profile. For standard deviation c ≥ 3, the average degrees in the anatomical scenario were larger than those in the electrophysiological setting. Connectivity in all networks was sparse, even for standard deviation c = 10 the average degree was just slightly more than 4% of the 10,201 potential connections per neuron.

To evaluate consistency, the results in Fig. 3 were compared against 10 subsequent network realizations. The variability across these realizations was minimal, with a relative standard deviation under 0.4% for all data points. The single trial in Fig. 3 differed from the 10-trial mean by less than 0.5% across all cases. Correspondingly, the z-scores (defined in Methods) fell between 0 and 1 in 11 cases, and between 1 and 2 in two cases, demonstrating high consistency of average degree across network realizations.

**Fig. 4:**
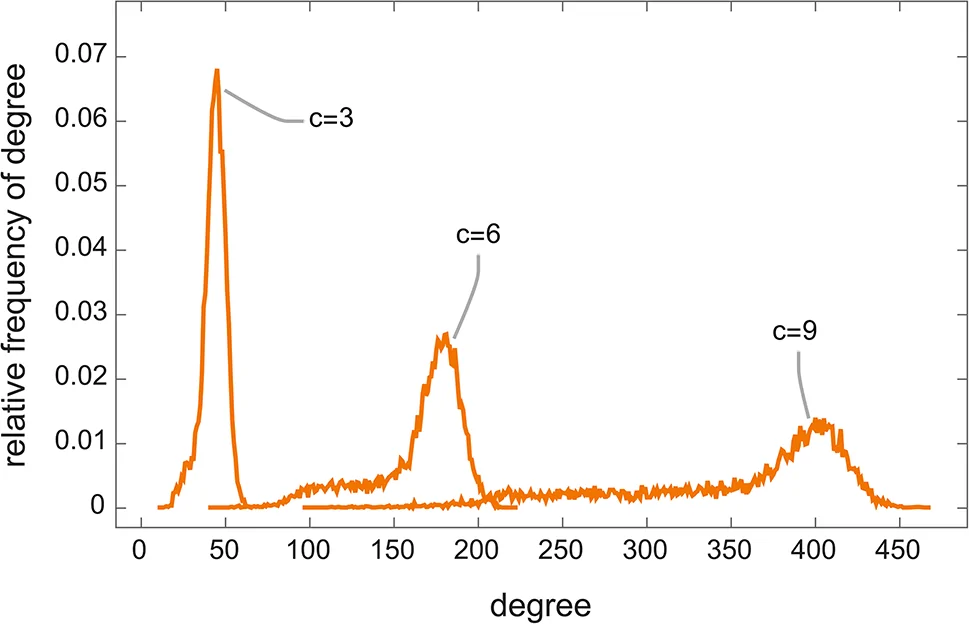
Degree distributions for distance dependent networks exemplified for standard deviations c = 3, c = 6 and c = 9

Figure 4 shows that degree distributions for distance-dependent networks broadened and became left-skewed as the standard deviation c of the Gaussians increased. This observation is supported by Kelley’s coefficient of skewness (* SK*) [40, 41], presented in Table 3. Kelley’s coefficient of skewness compares the 10th ($\:{P}_{10}$) and 90th ($\:{P}_{90}$) percentiles to the median ($\:{P}_{50}$):

$$\:SK=\frac{{P}_{90}+{P}_{10}-2{P}_{50}}{{P}_{90}-{P}_{10}}$$

As shown in Table 3, distribution skewness increases with larger standard deviations c, corresponding to wider Gaussian connectivity profiles. Wider profiles introduce edge effects at the boundaries of the quadratic monolayers, as boundary neurons form fewer connections than those in the center.

**Table 3:** Mean, median and Kelley’s skewness coefficient for different degree distributions

Standard deviation c of the Gaussian connectivity profile	mean	median	Kelley’s coefficient of skewness
c = 1	4.97	5	0
c = 2	19.48	20	-0.1111
c = 3	43.18	44	-0,1765
c = 4	75.47	78	-0.3333
c = 5	115.89	121	-0.4468
c = 6	164.37	173	-0.5068
c = 7	219.84	234	-0.5534
c = 8	282.39	303	-0.5652
c = 9	351.30	380	-0.5843
c = 10	426.30	461	-0.5555

The degree distributions in the configuration model are identical and not shown here.

### Local clustering coefficient

**Fig. 5:**
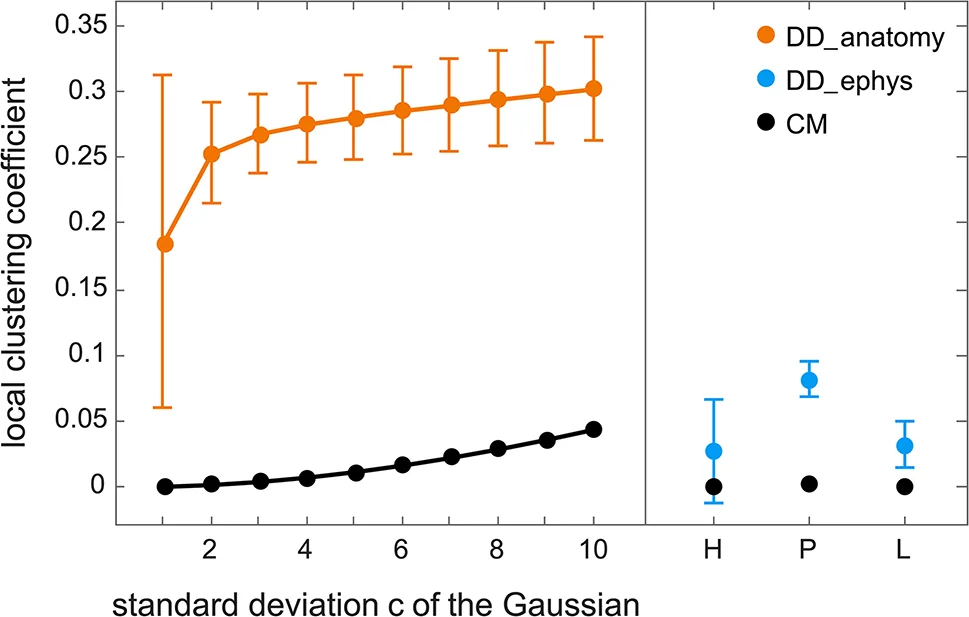
Average local clustering coefficients for distance dependent networks and the configuration model. Results for distance dependent networks are displayed in orange in the anatomical scenario (DD_anatomy) and in blue in the electrophyiological scenario (DD_ephys). Results for the configuration model (CM) are shown in black. The label “standard deviation c of the Gaussian” refers to the connectivity profiles in Fig. 1. The letters H, P and L are abbreviations for the studies by Holmgren et al. [25], Perin et al. [26] and Levy and Reyes [27]. Standard deviations are only shown if they are larger than the diameter of the dots

The local clustering coefficient is a measure of how well neighbors of a given neuron are connected. It is defined as the ratio of connected pairs of neighbors of a given neuron over all possibly existing connected pairs of neighbors. Figure 5 illustrates the average local clustering coefficients in 13 distance dependent networks and 13 networks generated by the configuration model.

Local clustering was much higher and more variable in distance dependent networks than in the configuration model. In the anatomical scenario local clustering coefficients were mostly between 0.25 and 0.3 in distance dependent networks, while they were < 0.045 in networks generated by the configuration model. Average local clustering coefficients in distance dependent networks were considerably larger in the anatomical scenario than in the electrophysiological setting, in the latter they did not exceed 0.082.

To evaluate consistency, Fig. 5 results were compared against 10 subsequent network realizations. Across both network types, average local clustering coefficients demonstrated high consistency.

For *distance-dependent* networks, relative standard deviations were under 1% (except one case at 2.39%), and single-trial values differed from 10-trial means by under 0.3% (except two cases at 1.95% and 2.30%). Corresponding * z*-scores remained low: 11 cases fell between 0 and 1 and 1 case between 1 and 2. The single outlier (* z*-score between 4 and 5 for * c=1* in the anatomical scenario) represented a tiny absolute deviation of 0.0042.

For *configuration model* networks, larger relative standard deviations (2.16–17.77%) and single trial-mean deviations (3.03–19.45%) were restricted to the four cases with the lowest average degrees. Because their baseline local clustering coefficients were near zero (0.0005–0.0019), these higher relative variations were minor in absolute terms. Otherwise, relative standard deviations and single trial-mean deviations remained below 1%, with * z*-scores falling between 0 and 1 (8 cases) and 1–2 (5 cases).

### Graph distance

Most of the 26 networks investigated here were strongly connected components, i.e. a directed path (with 1 or more links) existed between all possible pairs of neurons. There were only 6 exceptions: the distance-dependent networks and the configuration model with standard deviation c = 1 as well as the distance-dependent networks and the configuration model based on Holmgren et al. [25] and on Levy and Reyes [27]. In these exceptions the largest strongly connected component included between 98.77 and 99.99% of all neurons.

**Fig. 6:**
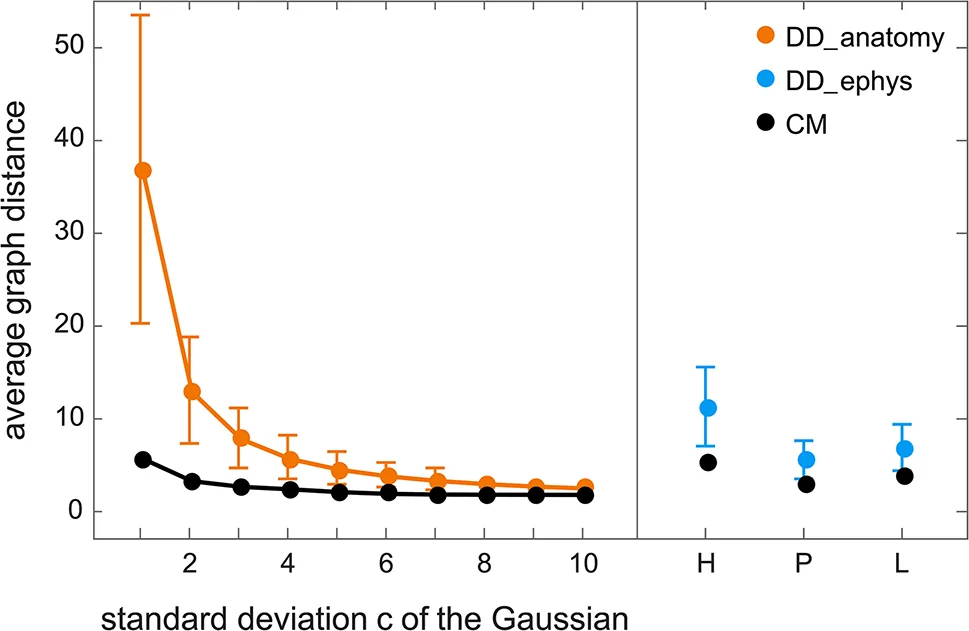
Average graph distances for distance dependent networks and the configuration model. Results for distance dependent networks are displayed in orange in the anatomical scenario (DD_anatomy) and in blue in the electrophyiological scenario (DD_ephys). Results for the configuration model (CM) are shown in black. The label “standard deviation c of the Gaussian” refers to the connectivity profiles in Fig. 1. The letters H, P and L are abbreviations for the studies by Holmgren et al. [25], Perin et al. [26] and Levy and Reyes [27]. Standard deviations are only shown if they are larger than the diameter of the dots

Graph distances denote geodesic distances between neurons, i.e. the shortest paths or the minimum numbers of links between two neurons. To determine the average graph distance the shortest directed paths between all pairs of neurons in the quadratic monolayer of 101 × 101 neurons were calculated. Figure 6 illustrates the average graph distance in 13 distance dependent networks and in 13 networks generated by the configuration model. In the 6 cases in which there was no single strongly connected component, average graph distances for the largest strongly connected component were calculated.

Average graph distances were larger and more variable in distance dependent networks than in the matching networks generated by the configuration model. In distance dependent networks of the anatomical scenario, the average graph distances decreased steeply from 36.76 to 5. 82 between standard deviations c = 1 and c = 4, for standard deviations c ≥ 5 the decline was much more gradual from 4.67 to 2.65. For standard deviations between c = 2 and c = 4, average graph distances of distance dependent networks were similar in both the anatomical and the electrophysiological setting, ranging between 13.03 and 5.82. There was only a small variation of average graph distances between 5.84 and 1.96 in the networks generated by the configuration model.

To evaluate consistency, Fig. 6 results were compared against 10 subsequent network realizations. Across both network types, average graph distances demonstrated high consistency.

For *distance-dependent* networks, relative standard deviations were under 0.7% across all cases, and single-trial values differed from 10-trial means by under 0.4%. Corresponding * z*-scores remained low: 7 cases fell between 0 and 1, 4 cases between 1 and 2, and 2 cases between 2 and 3.

For *configuration model* networks, relative standard deviations were under 0.3% across all cases, with single-trial values differing from 10-trial means by under 0.3%. Corresponding * z*-scores were similarly low: 9 cases fell between 0 and 1, 3 cases between 1 and 2, and 1 case between 2 and 3.

### Cliques

Cliques are subsets of neurons in which all neurons are adjacent. In this study this meant that there was a direct path from each neuron to every other neuron of the clique. Only maximal cliques were considered, i.e. cliques which could not be enlarged by adding another neuron. Cliques were required to include at least 3 neurons.

**Fig. 7:**
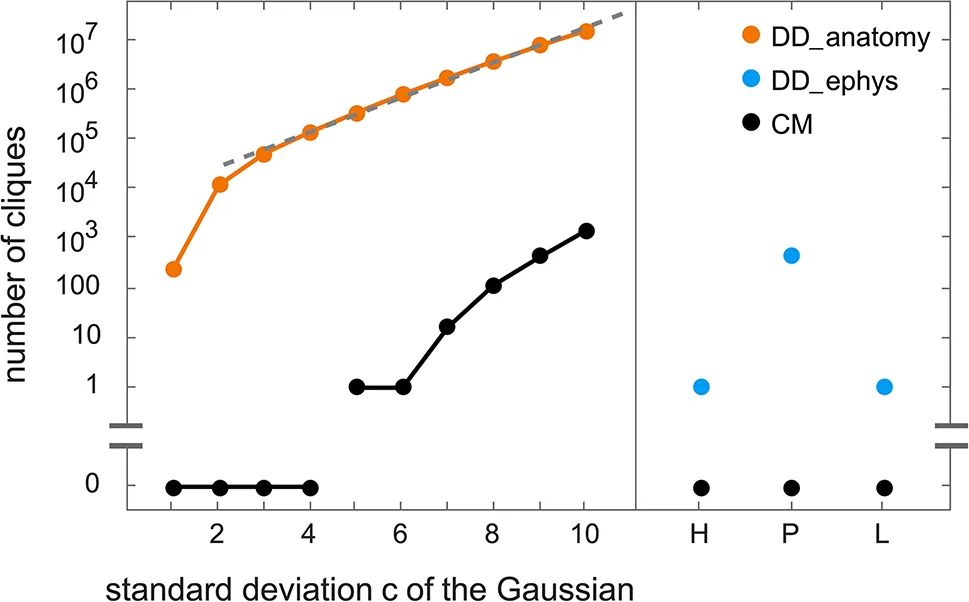
Numbers of cliques comprising at least 3 neurons for distance dependent networks and the configuration model. Results for distance dependent networks are displayed in orange in the anatomical scenario (DD_anatomy) and in blue in the electrophyiological scenario (DD_ephys). Results for the configuration model (CM) are shown in black. The label “standard deviation c of the Gaussian” refers to the connectivity profiles in Fig. 1. The letters H, P and L are abbreviations for the studies by Holmgren et al. [25], Perin et al. [26] and Levy and Reyes [27]. The increase in the number of cliques in the electrophysiological scenario can be approximated by a straight line for c ≥ 3 (dashed gray line; $\:\mathrm{log}\left(y\right)=0.351x+3.763$; $\:{R}^{2}=0.997$). Note the logarithmic scale on the vertical axis, which includes an axis break between 0 and 1 as zero cannot be plotted on a logarithmic scale

Figure 7 illustrates the numbers of cliques thus defined in both distance dependent networks and in networks generated by the configuration model.

Cliques were present in all distance dependent networks. In the configuration model cliques were only found in the anatomical scenario, provided the number of contacts was relatively high (standard deviation c ≥ 5 for the matching distance dependent networks). The number of cliques with at least 3 neurons was small, i.e. between 1 and 1479, in distance dependent networks of the electrophysiological scenario and in the configuration model. In contrast, cliques occurred much more frequently in distance dependent networks of the anatomical scenario. In the logarithmic plot of Fig. 7, the increase of the number of cliques could be approximated by a straight line for c ≥ 3 (log(y) = 0.351 x + 3.763; R^2^ = 0.997, indicating an excellent fit). This suggests an exponential rise to more than 1.6 × 10^7^ cliques for c = 10.

To evaluate consistency, Fig. 7 results were compared against 10 subsequent network realizations. Across both network types, clique counts demonstrated high consistency, with higher relative variation occurring only when baseline counts were small.

In *distance-dependent* networks, two cases (H and L) had only 1 clique in Fig. 7, ranging from 0 to 3 across realizations. Two other cases (c = 1 and P) had under 1,000 cliques, yielding relative standard deviations of 3.79% and 6.68%, with single-trial deviations from 10-trial means of 1.58% and 17.51%. For all other cases (clique counts > 10,000), relative standard deviations were below 1% and trial-mean differences under 0.6%. Corresponding z-scores for cases with over 1 clique were consistently low (10 cases between 0 and 1; 1 case between 2 and 3).

In *configuration model* networks, nine cases in Fig. 7 (c = 1 to c = 6, H, P, and L) contained 0 or 1 clique, ranging from 0 to 6 across realizations (strictly 0 in 6 cases, 0–2 in 2 cases, and 1–6 in 1 case). In the remaining four cases (c = 7 to c = 10), clique counts ranged between 17 and 1,540, with relative standard deviations between 4.07% and 20.98% and trial-mean differences between 3.09% and 35.36%. Corresponding z-scores remained low, with 3 cases falling between 0 and 1 and 1 case between 1 and 2.

**Fig. 8:**
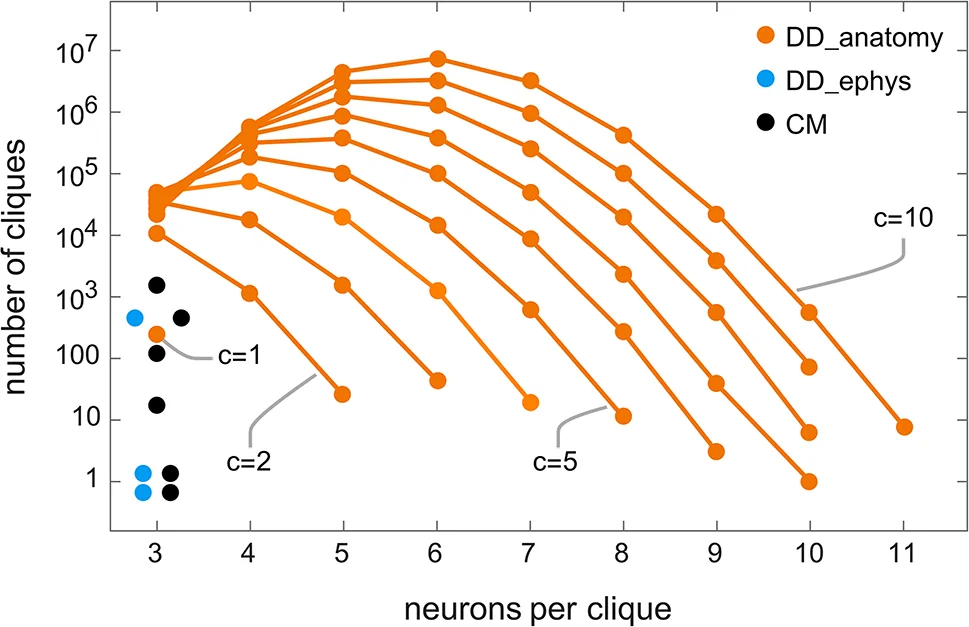
Numbers of cliques with specific numbers of neurons. Results for distance dependent networks are displayed in orange in the anatomical scenario (DD_anatomy) and in blue in the electrophyiological scenario (DD_ephys). Results for the configuration model (CM) are shown in black. The standard deviation c relates to the Gaussian connectivity profiles in Fig. 1. Note the logarithmic scale on the vertical axis

Figure 8 illustrates how many neurons were included in the cliques.

It is shown that the number of neurons per clique did not exceed 3 in distance dependent networks of the electrophysiological scenario and networks generated by the configuration model. In distance dependent networks of the anatomical scenario, more than 3 neurons per clique were possible for c ≥ 2. For c ≥ 4, cliques including more than 3 neurons prevailed. Up to 11 neurons per clique were reached for c = 10.

**Fig. 9:**
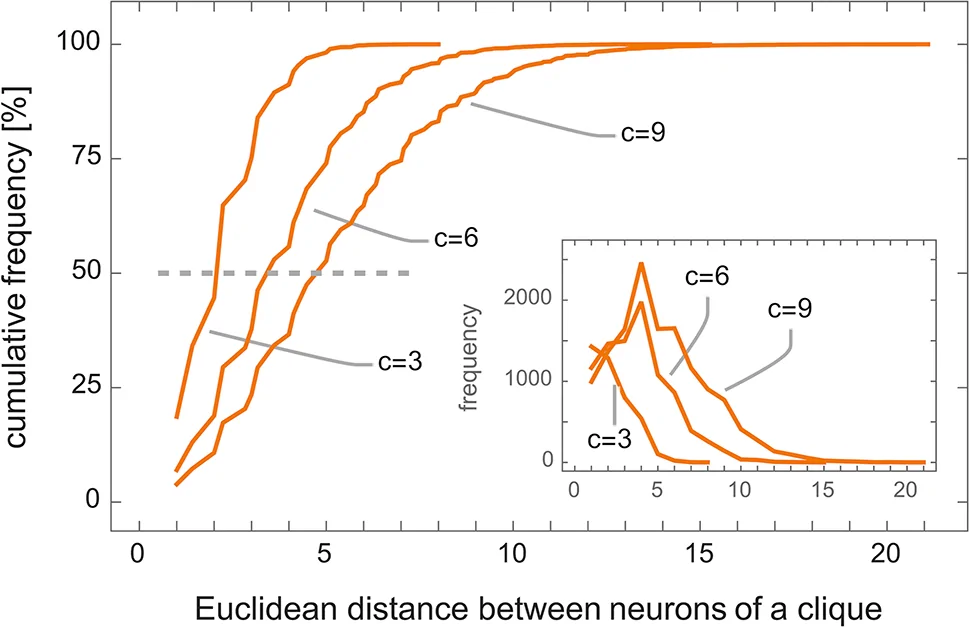
Cumulative (main panel) and individual (inset) frequencies of Euclidean distances between the neurons of a clique. Results are shown for standard deviations c = 3, c = 6 and c = 9 of the anatomical scenario. The horizontal grey dashed line marks the median of the cumulative frequency distributions. Median distances are 2.24 (c = 3), 3.61 (c = 6) and 5.00 (c = 9). Across all cases, the minimum distance between neurons of a clique is 1. Maximum distances are 8.0 (c = 3), 15.3 (c = 6) and 21.1 (c = 9)

To describe the spatial dimensions of cliques, randomly selected samples of 1000 cliques were investigated for each of the connectivity profiles with standard deviations c = 3, c = 6 and c = 9. Euclidean distances between the neurons of these cliques were calculated using Pythagoras’ theorem, their cumulative and individual frequencies being illustrated in Fig. 9.

It can be inferred from Fig. 9 that distances between neurons in a clique tend to be short (precise values for minimum, median and maximum distances are given in the legend to Fig. 9) as expected from the relatively high local clustering coefficient (Fig. 5). This indicates that there is a tendency in distance dependent networks to form highly localized, strongly connected groups of neurons.

### Euclidean distances between directly connected neurons

**Fig. 10:**
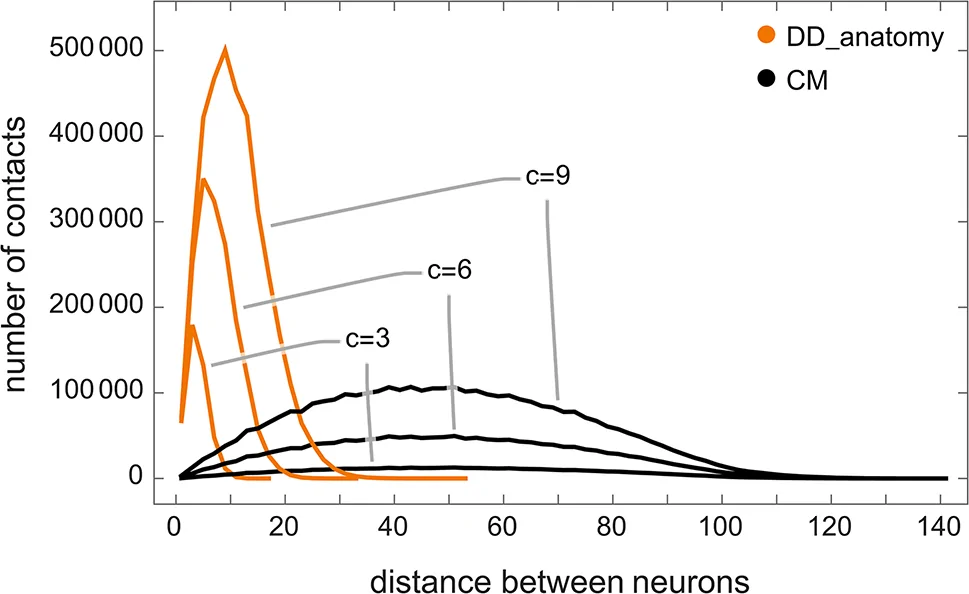
The frequency of specific Euclidean distances between neurons in distance dependent networks and the configuration model exemplified for standard deviations c = 3, c = 6 and c = 9. Results for distance dependent networks in the anatomical scenario are displayed in orange (DD_anatomy), results for the configuration model (CM) are shown in black. The bin size is 2

Figure 10 illustrates the frequency of specific Euclidean distances between two directly connected neurons. Distances were calculated using Pythagoras’ theorem. Euclidean distances between directly connected neurons should not be confounded with the graph distances discussed above. The latter denote geodesic distances between neurons, i.e. the shortest paths or the minimum numbers of links between two neurons.

It is shown that Euclidean distances between directly connected neurons tend to be short in distance-dependent networks, the peak of the distance distributions being below 10 base units. In contrast, Euclidean distances between neurons tend to be much larger in networks generated by the configuration model. Distance distributions are relatively flat with peaks around 40 to 50 base units.

### Cost of connectivity

Two factors seem to be important for the costs of a network.

1. The number of connections. More connections demand more cable and more expenditure for the maintenance of the network, e.g. caused by the need for larger amounts of neurotransmitters.
2. The distance between connected neurons. A larger distance between neurons means that more cable is required for the construction of the network and more energy has to be invested for its maintenance, e.g. for the axonal transport of neurotransmitters from the cell body to the presynaptic sites.

**Fig. 11:**
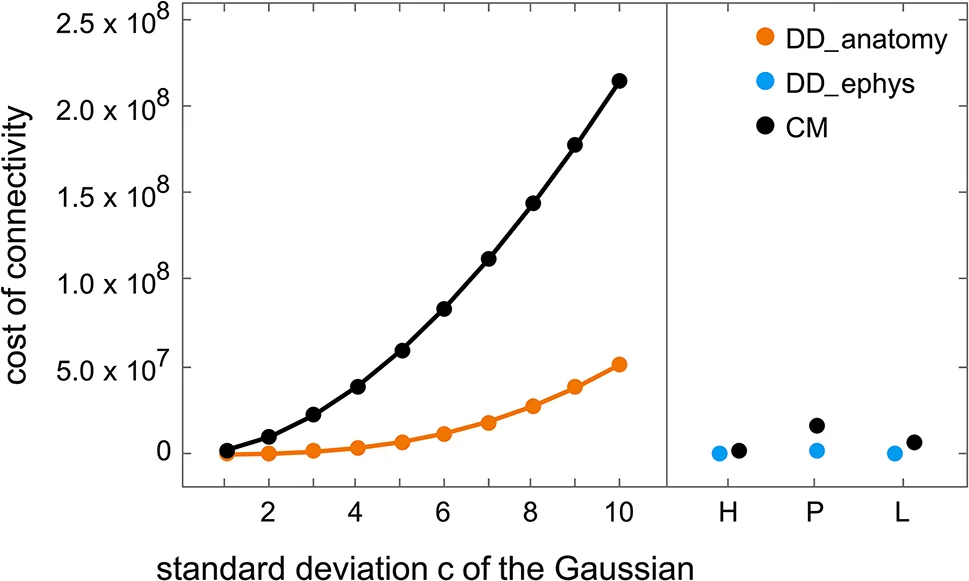
Total cost of connectivity calculated by multiplying the frequency of specific Euclidean distances between connected neurons with these distances. Results for distance dependent networks are displayed in orange in the anatomical scenario (DD_anatomy) and in blue in the electrophyiological scenario (DD_ephys). Results for the configuration model (CM) are shown in black. The label “standard deviation c of the Gaussian” refers to the connectivity profiles in Fig. 1. The letters H, P and L are abbreviations for the studies by Holmgren et al. [25], Perin et al. [26] and Levy and Reyes [27]

Here, the number of connections at specific Euclidean distances between neurons multiplied by these distances was used as a measure for the cost efficiency of a network. The sum of these calculations for each of the 26 networks reflects the total cost of connectivity and is illustrated in Fig. 11.

It is shown that the cost of connectivity is always lower in distance dependent networks than in networks generated by the configuration model due to the shorter connections in distance dependent networks (cf. Figure 10). In the anatomical scenario the cost of connectivity increases disproportionately with increasing standard deviation c. This is mainly due to the increasing number of connections with increasing standard deviation c, as shown in Fig. 4. In distance dependent networks the rising costs are also caused by the tendency to form higher proportions of more distant connections as illustrated by the connectivity profiles in Fig. 1 and the results in Fig. 10.

To evaluate consistency, Fig. 11 results were compared against 10 subsequent network realizations. Across both network types, the cost of connectivity demonstrated high consistency.

For *distance-dependent* networks, relative standard deviations were under 0.6% across all cases, and single-trial values differed from 10-trial means by under 0.8%. Corresponding z -scores remained low: 8 cases fell between 0 and 1, 4 cases between 1 and 2, and 1 case between 2 and 3.

For *configuration model* networks, relative standard deviations were under 0.5%, with single-trial values differing from 10-trial means by under 0.8%. Corresponding z-scores were similarly low: 10 cases fell between 0 and 1 and 3 cases between 1 and 2.

## Discussion

### The approach of this study

The networks investigated in this study were reduced to a radically simplified form. Only elementary network characteristics were considered, namely the numbers of pyramidal neurons involved, their layout in a quadratic monolayer and the rules by which the neurons were connected. The focus was on local cortical connectivity. If the quadratic monolayer of 101 × 101 pyramidal neurons was transferred into mouse cortex, the area covered would be about 2.34 × 2.34 mm^2^ (see Table 1). Intrinsic horizontal patchy connections [42–45], cortico-cortical connections via the white matter and projections from pieces of gray matter outside the cortex were disregarded. Moreover, the inhibitory non-pyramidal neurons were not considered.

The reduction to elementary network characteristics inevitably entailed the omission of some important aspects of cortical connectivity. Below is a list of factors concerning synapses and the heterogeneity of the cortex that were abstracted away, although their potential relevance is evident:

- The formation of synapses during cortical development.
- The number of synapses per connection.
- The strength of synapses shaped by learning processes.
- The cortical layering perpendicular to the cortical surface.
- The parcellation of the cortex into areas.
- The distinction between different subtypes of pyramidal neurons.
- The tendency of functionally similar neurons to be preferentially connected [46, 47].
- The functional topography of the cortex, e.g., the barrel cortex of somatosensory areas or ocular dominance and orientation columns in the visual cortex.

To justify the simplifying assumptions made here, it may be pointed out that there is a long tradition of exploring cortical connectomes in terms of statistics and geometry [1, 48, 49]. Combining this approach with the tools of network science [34] has proven highly effective [45, 50, 51].

### Spatial relationships between neurons matter

For each of the 13 distance dependent networks investigated here a matching network generated by the configuration model was studied. Spatial relations between neurons which define distance-dependent networks are disregarded in the configuration model. Distance-dependent networks and the corresponding networks of the configuration model were identical in some respects, concerning the in-degrees and out-degrees of each neuron, the overall number of connections and the degree distributions. Otherwise, distance dependent networks and the configuration model were very different. This applied to the local clustering coefficient (Fig. 5), the average graph distance or path length (Fig. 6), the numbers and sizes of cliques (Figs. 7 and 8), the Euclidean distance between connected neurons (Fig. 10) and the cost of connectivity (Fig. 11). In comparison, distance dependent networks were much more clustered, i.e. there was a focus on spatially localized wiring. This was reflected by the much larger propensity to form highly localized cliques in distance dependent networks. On the other hand, average graph distances were shorter in the configuration model. This was caused by a higher share of longer connections as revealed by the larger Euclidean distances between two connected neurons. Longer connections imply that more distant regions of the networks could be more easily reached. However, they mean also that the cost of connectivity was relatively large in the configuration model compared to distance dependent networks.

Both network types investigated here were random in the sense that connections were formed in a probabilistic way. However, the underlying probabilistic connectivity rule influenced the network structure enormously. Obviously, spatial relationships between neurons play a critical role.

The question as to whether connections in the cortex are random or specific has often been raised [e.g. 1, 7, 20]. The present findings indicate that the arguments in this discussion could be strengthened by explicitly stating which type of randomness is implied. Cortical wiring is strongly influenced by the geometry of axonal and dendritic trees [3, 30, 52]. This suggests that random networks disregarding spatial relations between neurons are quite unrealistic as a model of cortical connectivity. Instead, distance dependent networks which incorporate knowledge about the shapes of neuronal ramifications may serve as an appropriate benchmark in discussions about specificities of cortical wiring.

### Differences between the anatomical and electrophysiological scenarios

An important difference between the anatomical and electrophysiological scenarios was that the numbers of connections tended to be much higher in the anatomical setting (Fig. 4). This was certainly caused by the higher connection probabilities for neighboring neurons in the anatomical scenario. While the connection probability for distance 0 was set to 0.8 in the anatomically inspired networks, it ranged between 0.08 and 0.23 in the networks based on the studies by Holmgren et al. [25], Perin et al. [26] and Levy and Reyes [27]. The higher connection probability for neighboring neurons can also explain the higher local clustering in the anatomical scenario (Fig. 5).

The most striking difference between the anatomical and electrophysiological scenarios concerned the formation of cliques. In the anatomical scenario, the number of cliques with at least 3 constituents was > 10^4^ for standard deviations c ≥ 2 and increased exponentially to more than 1.6 × 10^7^ cliques for c = 10. In contrast, the number of cliques was low in the electrophysiological scenario, just 1 for the networks based on Holmgren et al. [25] and Levy and Reyes [27], 448 for the network based on Perin et al. [26]. Moreover, cliques with more than 3 members did not occur in the electrophysiological scenario, while they predominated in the anatomical scenario with up to 11 members per clique.

It is generally assumed that highly connected groups of neurons, such as Hebbian cell assemblies, are the functional building blocks of the cerebral cortex [1, 49, 53, 54]. It is unlikely that such neuronal assemblies can be simply equated with cliques, the definition of cliques being too restrictive. However, communication between cortical neurons is not wireless. Functional connectivity can only occur when anatomical connections are present. From this perspective, the networks based on electrophysiologically obtained connectivity rules seem to be quite disadvantageous, since the structural basis for the formation of highly connected groups of neurons is so weak.

The large discrepancy between anatomical and electrophysiological studies, as far as the connection probability between neighboring neurons is concerned, is certainly unsatisfactory. Considering the present findings, electrophysiological studies might underestimate connection probabilities, selecting strong connections mediated by multiple synapses while weaker, monosynaptic connections remain undetected. More evidence would be helpful, e.g., by investigating the connection probability of randomly selected, adjacent neurons by combined light and electron microscopy.

### Similarity of the anatomical and electrophysiological scenarios

The above paragraph highlighted differences between the anatomical and electrophysiological scenarios concerning the numbers of synapses, the local clustering coefficient, and the formation of cliques. The main factor driving these differences was likely to be the connection probability at distance 0 between neurons: it was high at 0.8 in the anatomical scenario and low between 0.08 and 0.23 in the electrophysiological scenario.

The Gaussian connectivity profiles in distance dependent networks are not only determined by the connection probability at distance 0, but also by the standard deviation c. In this respect it is noteworthy that average graph distances or path lengths were relatively similar in the anatomical and electrophysiological scenarios for standard deviations c ≥ 2 (Fig. 6).

When expressing the Gaussian connectivity profiles in Fig. 2 in the form of Eq. (1), the following values for the standard deviation c ensue (see Table 2): c = 3.48 in Holmgren et al. [25], c = 4.89 in Perin et al. [26] and c = 4.88 in Levy and Reyes [27]. These values are remarkably similar although they are derived from different species, cortical areas, and layers.

**Fig. 12:**
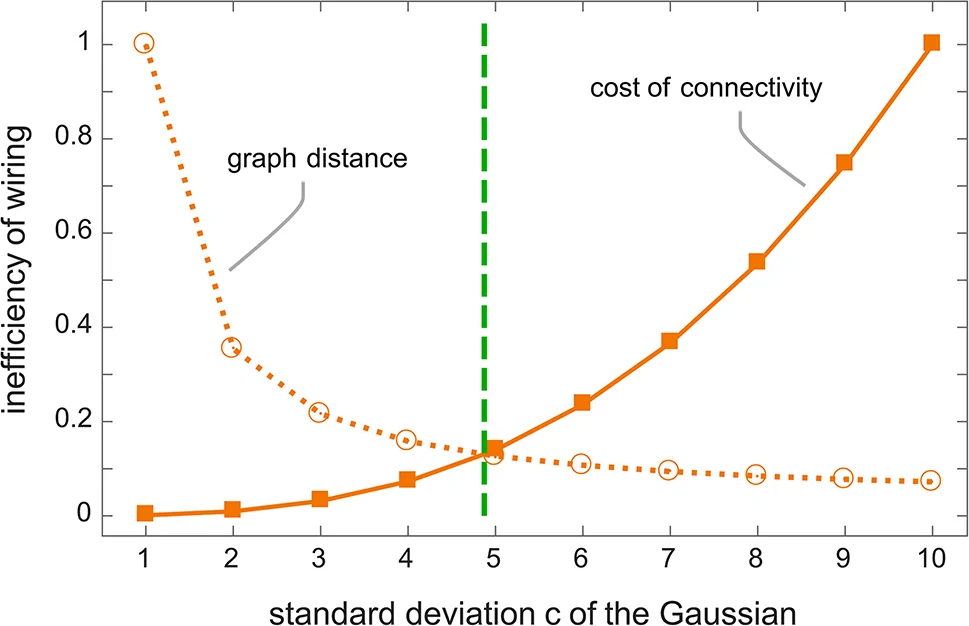
Inefficiency of wiring dependent on the standard deviation c of the Gaussian connectivity profiles in Fig. 1. Average graph distances from Fig. 6 (open circles, dotted line) and of the costs of connectivity from Fig. 11 (filled squares, solid line) were standardized by setting their highest values to 1. High values on the vertical axis were considered as unfavorable indicating inefficient wiring. The intersection of both lines indicated by the vertical green dashed line is at c = 4.87, the wiring inefficiency being at 0.13

In the anatomical scenario 10 Gaussian connectivity profiles were considered, the standard deviations c ranging between 1 and 10 (Fig. 1). The question arises if there is an optimum standard deviation c in the anatomical scenario. To this end, average graph distances and the cost of connectivity were placed together in one graph, the highest values of both parameters standardized to 1 to enable comparison (Fig. 12). In such a representation, high values on the vertical axis correspond to inefficiency of wiring assuming that high values for path lengths and costs of connectivity are unfavorable.

Figure 12 illustrates that the average graph distance decreases steeply for small values of c, while the cost of connectivity rises disproportionately for higher values of c. There is obviously a tradeoff between the two parameters. The intersection between both lines highlighted by the vertical green dashed line is considered as the optimum, since the inefficiency of wiring is relatively low at 0.13 in both cases. The two lines intersect at c = 4.87, which is basically identical with the standard deviations c of the connectivity profiles obtained by Perin et al. [26] and Levy and Reyes [27].

### Hypothesis about a general rule of local connectivity between pyramidal neurons

Gaussian connectivity profiles in distance dependent networks as defined by Eq. (1) are determined by just two parameters: the connection probability at Euclidean distance 0 between neurons and the standard deviation c of the Gaussian.

It was shown that the low connection probabilities found in electrophysiological studies led to disadvantageous network characteristics. In particular, the tendency to form highly localized, strongly connected neuronal ensembles was low. In this respect, the higher connection probability of 0.8 for neurons at Euclidean distance 0 based on anatomical evidence [3–5] appeared more alluring (Figs. 7 and 8).

As to the standard deviation c of Gaussian connectivity profiles anatomical and electrophysiological evidence seemed much more convergent. Despite being derived from different species, cortical areas and layers, c was virtually identical at 4.89 and 4.88 in Perin et al. [26] and Levy and Reyes [27]. The value obtained by Holmgren et al. [25] was not far off with c = 3.48. The completely independent statistical and geometrical reasoning in this study lead to an optimum standard deviation at c = 4.87 (Fig. 12), i.e. a value which was basically identical with the estimates obtained by Perin et al. [26] and Levy and Reyes [27].

Consequently, a hypothesis of a general rule for local connectivity between pyramidal neurons in the cerebral cortex can be proposed, based on Eq. (1) used in this study:

  

1 $$\:{\mathrm{p}=\mathrm{a}\:\mathrm{e}}^{\frac{{-\mathrm{x}}^{2}}{{2\mathrm{c}}^{2}}}$$

where * p* is the connection probability, * a* the connection probability at Euclidean distance 0 between neurons, * x* the Euclidean distance between neurons and $\:c\:$the standard deviation.

Following the reasoning above, it is assumed that a = 0.8 [5] and c = 4.88, i.e. the mean of the values obtained for Perin et al. [26], Levy and Reyes [27] and the anatomical scenario of this study (see Fig. 12).

In this formulation, the shortest distance between two pyramidal neurons along the edge of the quadratic monolayer used here is the base unit 1. This can be easily transferred to the dimensions of specified cortical layers of a given cortical area by using the volume density of neurons per mm^3^ which is generally readily available, and by bearing in mind that pyramidal neurons account for 85% of all neurons [1]. According to the argument made in Table 1, the shortest distance d between two pyramidal neurons along the edge of a quadratic monolayer can be expressed in µm as follows:

2 $${\rm{d = 1000/}}\root {\rm{3}} \of {{\rm{0}}{\rm{.85}}\,{\rm{v}}}$$

where v is the volume density of neurons per mm^3^ in a specified layer of a given cortical area.

By multiplying c of formula (1) with d of formula (2), formula (1) can then be converted from its standardized version (using the base unit 1 for Euclidean distances between neurons) to the dimensions of real cortices.

### Is the general rule for local cortical connectivity reasonable?

The question arises as to whether the general rule of local connectivity between pyramidal neurons proposed here makes sense. To check this, the connectivity rule is applied to mouse cortex involving as an independent parameter the volume density of synapses per mm^3^.

In mouse cortex, the number of synapses per mm^3^ is 7 × 10^8^, 75% of which are formed between pyramidal neurons [1, 36]. We consider a piece of cortex with the thickness of a typical cortical layer. To this end, 20 of the monolayers of 101 × 101 pyramidal neurons were stacked onto each other. Using the numbers from Table 1, the volume of this piece of mouse cortex is 2.336 × 2.336 × 0.444 mm^3^ = 2.423 mm^3^. Thus, the number of synapses between pyramidal neurons in this volume is 7 × 10^8^ × 0.75 × 2.423 = 1.272 × 10^9^.

This estimate is compared with the numbers obtained by applying formula (1) with a connection probability at Euclidean distance 0 between neurons of a = 0.8 and a standard deviation of the Gaussian connectivity profile of c = 5. In this scenario, the average out-degree of each pyramidal neuron in the quadratic monolayer of 101 × 101 neurons was 115.89 (Fig. 4). Thus, all neurons in the quadratic monolayer generate 115.89 × 101 × 101 = 1.182 × 10^6^ connections. It is assumed for the stack of 20 monolayers considered above that the connectivity rule is the same within and between monolayers. This yields 1.182 × 10^6^ × 20 × 20 = 4.728 × 10^8^ connections in the whole stack of 20 monolayers. Some of these connections are mediated by more than 1 synapse, i.e. the above estimate for the total number of connections has to be revised upwards. Lübke et al. [5] showed in a combined light and electron microscopic study that pyramidal neurons in layer 5 of rat somatosensory cortex formed on average 2.3 synapses with itself. This corresponds well to the estimate for directly adjacent neurons given by Hellwig [3] who also provided evidence that the average number of contacts decreases with increasing Euclidean distance between neurons. Thus, it can be concluded that the average number of synapses between pyramidal neurons in a local volume of cortex ranges predominantly between 1 and 2, as a rough estimate we assume 1.5. Multiplying the number of 4.728 × 10^8^ connections calculated above with a factor of 1.5 would yield 7,092 × 10^8^ synapses in a stack of 20 monolayers of 101 × 101 pyramidal neurons. This is slightly more than 50% of the overall number of synapses between pyramidal neurons estimated above.

The synapses in a piece of cortex are not only generated by the neurons whose cell bodies reside within this volume. Neurons outside the volume contribute as well, i.e. neurons in the same layer but outside the piece of cortex under consideration, neurons in other layers, neurons in other cortical areas and neurons in parts of the central nervous system outside the cortex. It is not precisely known what percentage of synapses in a piece of cortex is derived from neurons whose cell bodies are in this volume. Schüz et al. [55] found that the local axonal trees of pyramidal neurons account for 55–70% of their total axonal projections within the cortex. Thus, the above estimate that slightly more than 50% of synapses between pyramidal neurons are locally generated appears to be in a reasonable order of magnitude.

## Conclusion

The present study showed that different probabilistic connectivity rules lead to highly divergent network structures.

First, there is a sharp contrast between distance dependent networks and networks generated by the configuration model which disregard spatial relations between neurons. In comparison, distance dependent networks are much more locally clustered, form substantially larger numbers of highly localized, strongly connected ensembles of neurons and are more cost effective. Distance dependent networks incorporate information about the shape of neuronal ramifications. Thus, probabilistically wired distance dependent networks seem to be an appropriate benchmark in discussions about randomness versus specificity of cortical connectivity.

Second, the variability within the spectrum of distance dependent networks is large. Gaussian connectivity profiles in distance dependent networks are shaped by two parameters, the connection probability at Euclidean distance 0 between neurons and the standard deviation c of the Gaussian. Anatomical and electrophysiological studies led to heterogeneous results concerning the connection probability between neighboring neurons. Distance dependent networks inspired by anatomical data in which the connection probability between neighboring neurons is high seemed favorable, since network structures emerge which are characterized by highly localized, strongly connected groups of neurons (Figs. 7, 8 and 9). As to the standard deviation c of the Gaussian, anatomical and electrophysiological evidence seemed to be much more convergent. Figure 12 offered an explanation why a standard deviation slightly smaller than c = 5 might be adequate for Gaussian connectivity profiles: the tradeoff between path lengths and the costs of connectivity was minimized.

A simple, general rule for probabilistic local cortical connectivity was proposed. This rule is characterized by a Gaussian profile, utilizing a peak connection probability of a = 0.8 and a standard deviation of c = 4.88. Artificial neural networks built on this rule exhibit significant structural advantages over networks inspired by other experimentally derived wiring rules. Because this rule relies primarily on statistical principles and the geometric properties of axonal and dendritic branching, it offers an economical model for cortical wiring. Whether this statistical framework relates directly to the genetically encoded, innate wiring rules proposed by Zador [2] remains an open question for future investigation.

It was shown that the general rule for local cortical connectivity proposed here can be easily adapted to specific cortical layers of a given cortical area using the volume density of neurons per mm^3^, a number which is in general readily available. The networks based on this general rule of local cortical connectivity are characterized by the emergence of highly localized, strongly connected ensembles of neurons. Such network characteristics appear to be a good substrate for the formation of functional neuronal assemblies in which the structural layout can be modified and refined by learning processes. 

## Data Availability

The datasets generated and analyzed during the current study are available in the zenodo repository: https://doi.org/10.5281/zenodo.17455934.

## References

[1] Braitenberg V, Schüz A, Cortex: statistics and geometry of neuronal connectivity. Springer-Verlag Publishing; 1998.

[2] Zador AM. A critique of pure learning and what artificial neural networks can learn from animal brains. Nat Commun. 2019;10(1):3770. 31434893 10.1038/s41467-019-11786-6 PMC6704116

[3] Hellwig B. A quantitative analysis of the local connectivity between pyramidal neurons in layers 2/3 of the rat visual cortex. Biol Cybern. 2000;82(2):111–21. 10664098 10.1007/PL00007964

[4] van Pelt J, van Ooyen A. Estimating neuronal connectivity from axonal and dendritic density fields. Front Comput Neurosci. 2013;7:160. 24324430 10.3389/fncom.2013.00160 PMC3839411

[5] Lübke J, Markram H, Frotscher M, Sakmann B. Frequency and dendritic distribution of autapses established by layer 5 pyramidal neurons in the developing rat neocortex: comparison with synaptic innervation of adjacent neurons of the same class. J Neurosci. 1996;16(10):3209–18. 8627359 10.1523/JNEUROSCI.16-10-03209.1996 PMC6579140

[6] Kasthuri N, Hayworth KJ, Berger DR, Schalek RL, Conchello JA, Knowles-Barley S, Lee D, Vázquez-Reina A, Kaynig V, Jones TR, Roberts M, Morgan JL, Tapia JC, Seung HS, Roncal WG, Vogelstein JT, Burns R, Sussman DL, Priebe CE, Pfister H, Lichtman JW. Saturated Reconstruction of a Volume of Neocortex. Cell. 2015;162(3):648–61. 26232230 10.1016/j.cell.2015.06.054

[7] Motta A, Berning M, Boergens KM, Staffler B, Beining M, Loomba S, Hennig P, Wissler H, Helmstaedter M. Dense connectomic reconstruction in layer 4 of the somatosensory cortex. Science. 2019;366(6469):eaay3134. 31649140 10.1126/science.aay3134

[8] Thomson AM, West DC, Wang Y, Bannister AP. Synaptic connections and small circuits involving excitatory and inhibitory neurons in layers 2–5 of adult rat and cat neocortex: triple intracellular recordings and biocytin labelling in vitro. Cereb Cortex. 2002;12(9):936–53. 12183393 10.1093/cercor/12.9.936

[9] Yoshimura Y, Dantzker JL, Callaway EM. Excitatory cortical neurons form fine-scale functional networks. Nature. 2005;433(7028):868–73. 15729343 10.1038/nature03252

[10] Lefort S, Tomm C, Floyd Sarria JC, Petersen CC. The excitatory neuronal network of the C2 barrel column in mouse primary somatosensory cortex. Neuron. 2009;61(2):301–16. 19186171 10.1016/j.neuron.2008.12.020

[11] Hofer SB, Ko H, Pichler B, Vogelstein J, Ros H, Zeng H, Lein E, Lesica NA, Mrsic-Flogel TD. Differential connectivity and response dynamics of excitatory and inhibitory neurons in visual cortex. Nat Neurosci. 2011;14(8):1045–52. 21765421 10.1038/nn.2876 PMC6370002

[12] Avermann M, Tomm C, Mateo C, Gerstner W, Petersen CC. Microcircuits of excitatory and inhibitory neurons in layer 2/3 of mouse barrel cortex. J Neurophysiol. 2012;107(11):3116–34. 22402650 10.1152/jn.00917.2011

[13] Jouhanneau JS, Kremkow J, Dorrn AL, Poulet JF. In Vivo Monosynaptic Excitatory Transmission between Layer 2 Cortical Pyramidal Neurons. Cell Rep. 2015;13(10):2098–106. 26670044 10.1016/j.celrep.2015.11.011 PMC4688033

[14] Jouhanneau JS, Kremkow J, Poulet JFA. Single synaptic inputs drive high-precision action potentials in parvalbumin expressing GABA-ergic cortical neurons in vivo. Nat Commun. 2018;9(1):1540. 29670095 10.1038/s41467-018-03995-2 PMC5906477

[15] Petersen CC, Sakmann B, Petersen CC, Sakmann B. The excitatory neuronal network of rat layer 4 barrel cortex. J Neurosci. 2000;20(20):7579-86. 10.1523/JNEUROSCI.20-20-07579.2000 PMC6772852 11027217

[16] Beierlein M, Gibson JR, Connors BW. Two dynamically distinct inhibitory networks in layer 4 of the neocortex. J Neurophysiol. 2003;90(5):2987–3000. 12815025 10.1152/jn.00283.2003

[17] Sun QQ, Huguenard JR, Prince DA. Barrel cortex microcircuits: thalamocortical feedforward inhibition in spiny stellate cells is mediated by a small number of fast-spiking interneurons. J Neurosci. 2006;26(4):1219–30. 16436609 10.1523/JNEUROSCI.4727-04.2006 PMC6674555

[18] Bannister AP, Thomson AM. Dynamic properties of excitatory synaptic connections involving layer 4 pyramidal cells in adult rat and cat neocortex. Cereb Cortex. 2007;17(9):2190–203. 17116652 10.1093/cercor/bhl126

[19] Markram H, Lübke J, Frotscher M, Roth A, Sakmann B. Physiology and anatomy of synaptic connections between thick tufted pyramidal neurones in the developing rat neocortex. J Physiol. 1997;500(Pt 2):409–40. 9147328 10.1113/jphysiol.1997.sp022031 PMC1159394

[20] Song S, Sjöström PJ, Reigl M, Nelson S, Chklovskii DB. Highly nonrandom features of synaptic connectivity in local cortical circuits. PLoS Biol. 2005;3(3):e68. 15737062 10.1371/journal.pbio.0030068 PMC1054880

[21] Krieger P, Kuner T, Sakmann B. Synaptic connections between layer 5B pyramidal neurons in mouse somatosensory cortex are independent of apical dendrite bundling. J Neurosci. 2007;27(43):11473–82. 17959790 10.1523/JNEUROSCI.1182-07.2007 PMC6673216

[22] Brown SP, Hestrin S. Intracortical circuits of pyramidal neurons reflect their long-range axonal targets. Nature. 2009;457(7233):1133–6. 19151698 10.1038/nature07658 PMC2727746

[23] Mercer A, West DC, Morris OT, Kirchhecker S, Kerkhoff JE, Thomson AM. Excitatory connections made by presynaptic cortico-cortical pyramidal cells in layer 6 of the neocortex. Cereb Cortex. 2005;15(10):1485–96. 15647524 10.1093/cercor/bhi027

[24] Crandall SR, Patrick SL, Cruikshank SJ, Connors BW. Infrabarrels Are Layer 6 Circuit Modules in the Barrel Cortex that Link Long-Range Inputs and Outputs. Cell Rep. 2017;21(11):3065–78. 29241536 10.1016/j.celrep.2017.11.049 PMC5736017

[25] Holmgren C, Harkany T, Svennenfors B, Zilberter Y. Pyramidal cell communication within local networks in layer 2/3 of rat neocortex. J Physiol. 2003;551(Pt 1):139–53. 12813147 10.1113/jphysiol.2003.044784 PMC2343144

[26] Perin R, Berger TK, Markram H. A synaptic organizing principle for cortical neuronal groups. Proc Natl Acad Sci USA. 2011;108(13):5419–24. 10.1073/pnas.1016051108 PMC3069183 21383177

[27] Levy RB, Reyes AD. Spatial profile of excitatory and inhibitory synaptic connectivity in mouse primary auditory cortex. J Neurosci. 2012;32(16):5609–19. 22514322 10.1523/JNEUROSCI.5158-11.2012 PMC3359703

[28] Markram H, Muller E, Ramaswamy S, Reimann MW, Abdellah M, Sanchez CA, Ailamaki A, Alonso-Nanclares L, Antille N, Arsever S, Kahou GA, Berger TK, Bilgili A, Buncic N, Chalimourda A, Chindemi G, Courcol JD, Delalondre F, Delattre V, Druckmann S, Dumusc R, Dynes J, Eilemann S, Gal E, Gevaert ME, Ghobril JP, Gidon A, Graham JW, Gupta A, Haenel V, Hay E, Heinis T, Hernando JB, Hines M, Kanari L, Keller D, Kenyon J, Khazen G, Kim Y, King JG, Kisvarday Z, Kumbhar P, Lasserre S, Le Bé JV, Magalhães BR, Merchán-Pérez A, Meystre J, Morrice BR, Muller J, Muñoz-Céspedes A, Muralidhar S, Muthurasa K, Nachbaur D, Newton TH, Nolte M, Ovcharenko A, Palacios J, Pastor L, Perin R, Ranjan R, Riachi I, Rodríguez JR, Riquelme JL, Rössert C, Sfyrakis K, Shi Y, Shillcock JC, Silberberg G, Silva R, Tauheed F, Telefont M, Toledo-Rodriguez M, Tränkler T, Van Geit W, Díaz JV, Walker R, Wang Y, Zaninetta SM, DeFelipe J, Hill SL, Segev I, Schürmann F. Reconstruction and Simulation of Neocortical Microcircuitry. Cell. 2015;163(2):456–92. 26451489 10.1016/j.cell.2015.09.029

[29] Reimann MW, King JG, Muller EB, Ramaswamy S, Markram H. An algorithm to predict the connectome of neural microcircuits. Front Comput Neurosci. 2015;9:120. 26500529 10.3389/fncom.2015.00120 PMC4597796

[30] Udvary D, Harth P, Macke JH, Hege HC, de Kock CPJ, Sakmann B, Oberlaender M. The impact of neuron morphology on cortical network architecture. Cell Rep. 2022;39(2):110677. 35417720 10.1016/j.celrep.2022.110677 PMC9035680

[31] Peters A, Feldman ML. The projection of the lateral geniculate nucleus to area 17 of the rat cerebral cortex. I. General description. J Neurocytol. 1976;5(1):63–84. 1249593 10.1007/BF01176183

[32] Peters A. Thalamic input to the cerebral cortex. Trends Neurosci. 1979;2:183–5.

[33] Bollobás B. A probabilistic proof of an asymptomatic formula for the number of labelled regular graphs. Eur J Combin. 1980;1(4):311–6.

[34] Barabási AL. Network science. Cambridge: Cambridge University Press; 2016.

[35] Meyer HS, Wimmer VC, Oberlaender M, de Kock CP, Sakmann B, Helmstaedter M. Number and laminar distribution of neurons in a thalamocortical projection column of rat vibrissal cortex. Cereb Cortex. 2010;20(10):2277–86. 20534784 10.1093/cercor/bhq067 PMC2936806

[36] Schüz A, Palm G. Density of neurons and synapses in the cerebral cortex of the mouse. J Comp Neurol. 1989;286(4):442–55. 2778101 10.1002/cne.902860404

[37] Keller D, Erö C, Markram H. Cell Densities in the Mouse Brain: A Systematic Review. Front Neuroanat. 2018;12:83. 30405363 10.3389/fnana.2018.00083 PMC6205984

[38] Brodmann K. Vergleichende Lokalisationslehre der Großhirnrinde in ihren Prinzipien dargestellt auf Grund des Zellenbaues. Leipzig: Barth; 1909.

[39] Hellwig B. Structural characteristics of local cortical networks wired by distance dependent connectivity rules (dataset). Zenodo. 2025. 10.5281/zenodo.17455934. PMC13589859 42763393

[40] Kelley TL. Statistical method. Macmillan; 1923.

[41] Sharma JK. Business statistics. Vikas Publishing House; 2020.

[42] Rockland KS, Lund JS. Intrinsic laminar lattice connections in primate visual cortex. J Comp Neurol. 1983;216(3):303–18. 6306066 10.1002/cne.902160307

[43] Gilbert CD, Wiesel TN. Clustered intrinsic connections in cat visual cortex. J Neurosci. 1983;3(5):1116–33. 6188819 10.1523/JNEUROSCI.03-05-01116.1983 PMC6564507

[44] Binzegger T, Douglas RJ, Martin KA. Stereotypical bouton clustering of individual neurons in cat primary visual cortex. J Neurosci. 2007;27(45):12242–54. 17989290 10.1523/JNEUROSCI.3753-07.2007 PMC6673271

[45] Voges N, Guijarro C, Aertsen A, Rotter S. Models of cortical networks with long-range patchy projections. J Comput Neurosci. 2010;28(1):137–54. 19866352 10.1007/s10827-009-0193-z

[46] Lee WC, Bonin V, Reed M, Graham BJ, Hood G, Glattfelder K, Reid RC. Anatomy and function of an excitatory network in the visual cortex. Nature. 2016;532(7599):370–4. 27018655 10.1038/nature17192 PMC4844839

[47] Ding Z, Fahey PG, Papadopoulos S, Wang EY, Celii B, Papadopoulos C, Chang A, Kunin AB, Tran D, Fu J, Ding Z, Patel S, Ntanavara L, Froebe R, Ponder K, Muhammad T, Bae JA, Bodor AL, Brittain D, Buchanan J, Bumbarger DJ, Castro MA, Cobos E, Dorkenwald S, Elabbady L, Halageri A, Jia Z, Jordan C, Kapner D, Kemnitz N, Kinn S, Lee K, Li K, Lu R, Macrina T, Mahalingam G, Mitchell E, Mondal SS, Mu S, Nehoran B, Popovych S, Schneider-Mizell CM, Silversmith W, Takeno M, Torres R, Turner NL, Wong W, Wu J, Yin W, Yu SC, Yatsenko D, Froudarakis E, Sinz F, Josić K, Rosenbaum R, Seung HS, Collman F, da Costa NM, Reid RC, Walker EY, Pitkow X, Reimer J, Tolias AS. Functional connectomics reveals general wiring rule in mouse visual cortex. Nature. 2025;640(8058):459–69. 40205211 10.1038/s41586-025-08840-3 PMC11981947

[48] Sholl DA. The organization of the cerebral cortex. John Wiley; 1956.

[49] Abeles M, Corticonics. Neural Circuits of the Cerebral Cortex. Cambridge: Cambridge University Press; 1991.

[50] Sporns O, Zwi JD. The small world of the cerebral cortex. Neuroinformatics. 2004;2(2):145–62. 15319512 10.1385/NI:2:2:145

[51] Bullmore E, Sporns O. Complex brain networks: graph theoretical analysis of structural and functional systems. Nat Rev Neurosci. 2009;10(3):186–98. 19190637 10.1038/nrn2575

[52] Stepanyants A, Chklovskii DB. Neurogeometry and potential synaptic connectivity. Trends Neurosci. 2005;28(7):387–94. 15935485 10.1016/j.tins.2005.05.006

[53] Sejnowski TJ. The book of Hebb. Neuron. 1999;24(4):773–6. 10624941 10.1016/s0896-6273(00)81025-9

[54] Palm G, Knoblauch A, Hauser F, Schüz A. Cell assemblies in the cerebral cortex. Biol Cybern. 2014;108(5):559–72. 24692024 10.1007/s00422-014-0596-4

[55] Schüz A, Chaimow D, Liewald D, Dortenman M. Quantitative aspects of corticocortical connections: a tracer study in the mouse. Cereb Cortex. 2006;16(10):1474–86. 16357338 10.1093/cercor/bhj085

